# Supplementary material for: MetaRibo-Seq measures translation in microbiomes
Source: Nat Commun. 2020 Jun 29;11:3268. doi: 10.1038/s41467-020-17081-z (PMC7324362; doi:10.1038/s41467-020-17081-z)
Supplement: Supplementary file 10 — Supplementary Data 7 [file 41467_2020_17081_MOESM10_ESM.zip › File2/Confidence_VeryHigh_Taxonomy/386892_out.krona.html]

Javascript must be enabled to view this page.

members
magnitude
magnitudeUnassigned
count
unassigned
taxon
rank

386892\_out

22

2
22
superkingdom

22
phylum
1224

class
22
1236

91347
order
22

543
22
family

SRS077502\_contig\_number\_contig-100\_636.46865
1

genus
1
544

545

SRS971427\_contig\_number\_17797
species
1

561
20
genus

SRS140492\_contig\_number\_contig-100\_433.170331
1

species
19
562

SRS012273\_contig\_number\_15668SRS017191\_contig\_number\_19558SRS017521\_contig\_number\_50243SRS049402\_contig\_number\_16215SRS075947\_contig\_number\_16350SRS076876\_contig\_number\_50SRS077127\_contig\_number\_16541SRS077454\_contig\_number\_11141SRS098881\_contig\_number\_22642SRS105082\_contig\_number\_14409SRS142781\_contig\_number\_2897SRS143014\_contig\_number\_contig-100\_160.161SRS143423\_contig\_number\_contig-100\_7795.126079SRS143596\_contig\_number\_contig-100\_666.667SRS144219\_contig\_number\_contig-100\_264.265SRS144362\_contig\_number\_23911SRS146888\_contig\_number\_13856SRS146996\_contig\_number\_20275SRS147022\_contig\_number\_12932
